# Monitoring of Serial Presurgical and Postsurgical Changes in the Serum Proteome in a Series of Patients with Calcific Aortic Stenosis

**DOI:** 10.1155/2015/694120

**Published:** 2015-05-20

**Authors:** Kazumi Satoh, Kazuo Yamada, Tomoko Maniwa, Teiji Oda, Ken-ichi Matsumoto

**Affiliations:** ^1^Department of Biosignaling and Radioisotope Experiment, Interdisciplinary Center for Science Research, Organization for Research, Shimane University, Izumo, Shimane 693-8501, Japan; ^2^Department of Biochemistry, Faculty of Medicine, Shimane University, Izumo, Shimane 693-8501, Japan; ^3^Division of Cardiovascular and Thoracic Surgery, Department of Surgery, Faculty of Medicine, Shimane University, Izumo, Shimane 693-8501, Japan

## Abstract

*Background*. Comprehensive analysis of proteome differentially expressed in response to surgery or drug treatment is useful to understand biological responses to dispensed interventions. Here we investigated expression changes in sera of patients who suffered from calcific aortic stenosis (CAS), before and after surgery for aortic valve replacement. *Materials and Methods*. Sera obtained before and after surgery with depletion of highly abundant proteins were analyzed with iTRAQ labeling followed by nanoLC-MALDI-TOF/TOF-MS/MS. *Results*. Fifty-one proteins shared in five patients were identified with differential levels in postsurgical and presurgical sera. Finally, 16 proteins that show statistically significant levels in patients' sera compared with those in control sera (*P* < 0.05) were identified. Most of the identified proteins were positive acute-phase proteins. Among three proteins other than acute-phase proteins, we confirmed increased levels of antithrombin-III and zinc-*α*-2-glycoprotein in postsurgical sera by Western blot analysis using other CAS patients' sera. Furthermore, antithrombin-III and zinc-*α*-2-glycoprotein were not found among proteins with differential levels in postsurgical and presurgical sera of patients with aortic aneurysms that we identified in a previous study. *Conclusions*. The results indicated that antithrombin-III and zinc-*α*-2-glycoprotein would become unique monitoring proteins for evaluating pathophysiological and biochemical processes occurring before and after surgery for CAS.

## 1. Introduction

Calcific aortic stenosis (CAS) is the most common clinical problem of valve disease in elderly people, afflicting some of individuals by the age of 65 years [1]. The early asymptomatic stage of calcific aortic valve disease (CAD) is characterized by mild thickening of the valve without obstruction of left ventricular outflow, termed aortic valve sclerosis. However, after slow progression of the disease to a more advanced stage, namely, CAS, impaired leaflet motion with severe calcium deposition in the valve leaflet occurs, subsequently leading to limitation of blood flow through the valve [2].

The pathophysiological mechanisms underlying CAS remain obscure [3–5]. Until recently, CAS has been generally considered as a degenerative process induced by repetitive and substantial mechanical stress with passive calcium deposition to the valve leaflets over many years. However, recent evidence indicates that CAS is an active disease process associated with endothelial disruption, chronic inflammatory changes, lipid deposition, and osteogenic changes at the valve leaflets [3, 6]. Studies indicate that abnormal mechanical forces such as hypertension, increasing stretch, or shear stresses loaded to the valve leaflet might trigger a series of changes that lead to end-stage calcification in valve leaflets [7].

Diagnostic evaluation of CAD includes assessment of the presence of aortic valve disease, extent of calcification, and delineation of left ventricular size and function by echocardiography [3]. Since effective medical therapy for CAS has not so far been available, aortic valve replacement remains the main treatment option [4]. Therefore, identification of circulating biomarkers diagnostic for characterization of early stages before progression to a severe symptomatic stage of CAS is needed. A large number of studies on biomarkers for diagnosis or prognosis of CAS have been published [2]. Among them, Sainger et al. [8] provided promising biomarkers in the multistage process of CAD by showing associations of plasma levels of osteopontin, parathyroid hormone, and fetuin-A (also known as *α*-2-HS-glycoprotein) with the early stage of CAD and associations of plasma levels of N-terminal prohormone of brain natriuretic peptide (NT-proBNP) and asymmetric dimethyl arginine (ADMA) with aortic valve stenosis.

Recently, proteomics-based techniques have also been used for identification of biomarkers of diagnosis, prognosis, and/or therapy responses in the field of cardiovascular diseases [9–11]. Our group [12] reported serum proteins with differential levels in postsurgery and presurgery after removal of aneurysmal tissues in patients with abdominal aortic aneurysm (AAA) and thoracic aortic aneurysm (TAA), which we identified by quantitative proteome analyses using tandem mass spectrometry (MS/MS) with an isobaric tag for a relative and absolute quantitation (iTRAQ) labeling strategy [13]. In that study, kallistatin in both AAA and TAA patients and *α*-2-macroglobulin in TAA patients showed decreased levels in postsurgical sera, similar to those in control sera, compared with those in presurgical sera. The results indicated that kallistatin is a potential serum biomarker for both AAA and TAA, whereas *α*-2-macroglobulin is that for TAA.

The approach to compare serial protein profiles in one patient is based on the idea that if interindividual difference of the level of each protein is large within a patient group, it might obscure the differential level of the protein associated with pathological condition that occurs in individual patients. Therefore, comparison of serial protein profiles from one individual patient is useful for conquest of interindividual variability among each group [14, 15]. It was also expected that the postsurgical level of a certain protein would be restored to the same level as that in normal control sera after dispensed interventions, if the protein can become a candidate of biomarkers for the disease.

Apart from the advantage for discovery of clinical biomarkers, the use of a pair of presurgical and postsurgical sera from one individual may help to monitor serial changes in serum proteome that occur in response to interventions such as surgery and medication. This would lead to an understanding of the pathophysiological and biochemical processes underlying the interventions.

In this study, in order to reveal the distinct proteome in postsurgical sera compared with that in presurgical sera from patients undergoing surgery for CAS with an aortic valve replacement procedure, we performed proteomic analyses with presurgical and postsurgical sera from each patient using the iTRAQ labeling, followed by nanoliquid chromatography- (nanoLC-) matrix-assisted laser desorption ionization (MALDI) time-of-flight (TOF/TOF) tandem mass spectrometry (MS/MS) [16].

## 2. Materials and Methods

### 2.1. Patients and Blood Sampling

Blood samples were obtained after receiving approval from the Ethics Committee of Shimane University School of Medicine, and the collection was conducted at Shimane University Hospital. The patients provided written informed consent. Blood samples for proteomic analyses were collected at presurgery and postsurgery from 5 CAS patients (referred to as P1~P5) (4 males, 1 female; average age, 73.0 years) who underwent aortic valve replacement. Other presurgical and postsurgical blood samples for confirmation by Western blot analysis were collected from 14 CAS patients (P6~P19) (4 males, 10 females; average age, 78.9 years). Presurgical and postsurgical blood samples were taken at an average of 7.5 days before surgery and an average of 13.1 days after surgery, respectively. Comorbidities (Table 1) and prescribed drugs (Table 2) restricted to cardiovascular diseases, lipid metabolism disorders, and calcium and phosphorus metabolism disorders that may cause ectopic calcification are shown for the 19 enrolled CAS patients. Other prominent disorders were diabetes mellitus (21.1% of the patients), prostate hypertrophy (10.5%), and rheumatoid arthritis (10.5%). Baseline characteristics were similar in the P1~P5 group and P6~P19 group compared by using Fisher's extract test (*P* > 0.05). As control samples for proteomic analyses, blood samples were collected from 4 healthy volunteers (N1~N4) (2 males, 2 females; average age, 42.0 years) at two time points [first time point (*T*1) and second time point (*T*2)] with a 14-day interval. Control sera from 10 other volunteers (N5~N14) (7 males, 3 females; average age, 42.0 years) were also collected at one time point for Western blot analysis. Blood samples were centrifuged and the plasma layer was removed. Serum samples were stored at −80°C until use.

### 2.2. Immunodepletion of Abundant Serum Proteins

To remove the two most abundant serum proteins, albumin and immunoglobulin (IgG), an albumin and IgG depletion SpinTrap column was used according to the manual of the manufacturer (GE Healthcare, Buckinghamshire, UK) and according to our previous study [12]. Fifty *μ*L of serum from each of the patients and healthy individuals was treated. After removal of the abundant proteins, the serum sample was equilibrated with 50 mM triethylammonium bicarbonate (TEAB) (Sigma, Tokyo, Japan) using spin concentrators (Corning, Tokyo, Japan). The protein concentration was determined using a bicinchoninic acid (BCA) protein assay reagent (Thermo Fisher Scientific, Rockford, IL, USA).

### 2.3. Sample Preparation

Sample preparation was performed according to the manual of the manufacturer (AB Sciex, Foster, CA, USA) and according to our previous study [12]. Briefly, 125 *μ*g each of immunodepleted presurgical and postsurgical serum samples from a patient was denatured by sodium dodecyl sulfate (SDS) and reduced by [tris-(2-carboxyethyl)phosphine (TCEP)], followed by cysteine alkylation with methyl methanethiosulfonate (MMTS). Subsequently, each sample was digested by trypsin (AB Sciex). Each digest was labeled with a different iTRAQ tag by an iTRAQ Reagent Multiplex kit (AB Sciex). iTRAQ label 114 or 115 was used for labeling presurgical serum samples, and iTRAQ label 116 or 117 was used for postsurgical serum samples. Then the labeled presurgical and postsurgical samples were mixed. This process was done for each sample from 5 patients (P1~P5). For the control samples collected at two time points, the sample at *T*1 was labeled with iTRAQ label 114 or 115, while the sample at *T*2 was labeled with iTRAQ label 116 or 117. Subsequently, the labeled samples were mixed. This process was done for each sample from 4 healthy volunteers (N1~N4). Thereafter, each of combined samples was fractionated into 6 fractions by strong cation exchange (SCX) chromatography according to the manual of the manufacturer (AB Sciex). Then each fraction was desalted by a Sep-Pac C_18_ cartridge according to the manual of the manufacturer (Waters, Milford, MA, USA).

### 2.4. NanoLC and MS/MS Analyses

A DiNa nanoLC system (KYA Technologies, Tokyo, Japan) was used for further fractionating one fraction from SCX chromatography to 171 spots according to the manufacturer's manual and our previous report [12]. The fractionated peptide samples were mixed directly with a matrix [4 mg/mL *α*-cyano-4-hydroxycinnamic acid (CHCA), Wako, Osaka, Japan] and then spotted onto an Opti-TOF LC/MALDI 384 target plate (AB Sciex) using a Dina MaP fraction collector (KYA Technologies).

The spotted peptide samples were analyzed on a 5800 MALDI-TOF/TOF MS/MS Analyzer with TOF/TOF Series software (version 4.0) (AB Sciex) to obtain mass spectrometry (MS) and MS/MS data according to the manufacturer's instructions and our previous report [12]. Monoisotopic precursor selection for MS/MS was done by automatic precursor selection with an interpretation method using the DynamicExit Algorithm (AB Sciex). The peptide data from 5800 MALDI TOF/TOF MS/MS were analyzed with ProteinPilot 3.0 software using the Paragon protein database search algorithm (AB Sciex) [17]. Search results were filtered for global false discovery rate (FDR) of 5% employing a decoy search strategy utilizing a reverse database. Each MS/MS spectrum was searched against the database constructed by AB Sciex (version 20081216, 40,978 entries). The statistic method of iTRAQ analyses followed ProteinPilot software (AB Sciex).

### 2.5. Bioinformatic Analysis

The Panther system (version 8.1) (http://www.pantherdb.org/) was used for protein classification analyses [18]. Statistical analyses of protein classification were done with a statistical overrepresentation test using Panther software by Bonferroni correction for multiple testing. The annotations of proteins were obtained from the UniProt database (http://www.uniprot.org/) and appropriate literature. Hierarchical clustering analysis of the proteins with differential levels was performed using Genesis software provided by the Genesis team of the Institute for Genomics and Bioinformatics, Graz University of Technology (Graz, Austria) (http://genome.tugraz.at/) [19].

### 2.6. Western Blot Analysis

Western blot analyses were performed as described in our previous paper [20]. Albumin/IgG immunodepleted-serum samples for Western blot analyses of ceruloplasmin and fibronectin or crude sera for analyses of antithrombin-III, zinc-*α*-2-glycoprotein, and *α*-1B-glycoprotein were electrophoresed through sodium dodecyl sulfate-polyacrylamide gel (SDS-PAGE), and then the proteins were transferred onto Hybond ECL nitrocellulose membranes (GE Healthcare Japan, Hino, Japan). The amounts of albumin/IgG immunodepleted sera (*μ*g) and crude sera (*μ*L) for each analysis were as follows: ceruloplasmin (10 *μ*g), fibronectin (10 *μ*g), antithrombin-III (0.5 *μ*L), zinc-*α*-2-glycoprotein (0.05 *μ*L), and *α*-1B-glycoprotein (0.05 *μ*L). The membranes were reacted with rabbit polyclonal anti-fibronectin antibody (2,000-times dilution) (Sigma-Aldrich, St. Louis, MO, USA), rabbit polyclonal anti-ceruloplasmin antibody (1,000-times dilution) (Epitomics, Burlingame, CA, USA), rabbit monoclonal anti-antithrombin-III antibody (1,000-times dilution) (GeneTex, Hsinchu, Taiwan), rabbit polyclonal anti-zinc-*α*-2-glycoprotein (1,000-times dilution) (Sigma-Aldrich), and rabbit polyclonal anti-*α*-1B-glycoprotein antibody (1,000-times dilution) (Bioss, Woburn, MA, USA). Subsequently, the proteins on the nitrocellulose membranes were reacted with donkey IRDye 680-conjugated anti-rabbit IgG (H+L) (5,000 times dilution) (LI-COR, Lincoln, NE, USA), followed by visualization using the infrared imaging system Odyssey (LI-COR). The intensity of each band that reacted with a corresponding antibody was measured for densitometric analysis of each protein level. Data from triplicate experiments were analyzed for statistical significance by Student's* t*-test. Its significance was set with *P* < 0.05. Results are expressed as means ± standard error (SE).

## 3. Results

### 3.1. Proteomic Analyses of Serum Proteins with Differential Levels in Postsurgical Sera Compared with Those in Presurgical Sera of CAS Patients

Presurgical and postsurgical serum samples were obtained from five CAS patients (P1~P5) who underwent aortic valve replacement. Protein levels in postsurgical sera were compared with those in presurgical sera using iTRAQ labeling coupled to nanoLC-MALDI-TOF/TOF-MS/MS followed by ProteinPilot analysis. Relative quantitation by ProteinPilot analysis was based on statistical analysis [17]. We set global FDR to 5%. The average iTRAQ ratios of peptides in postsurgical sera to those in presurgical sera were calculated. Except for albumin, Ig family members and reversed sequences in FDR Database, 109 proteins with differential levels (postsurgical versus presurgical) were identified as proteins that appear at least once with an unused ProtScore of ≧2 (99% confidence) by analyses of sera from the five patients, of which 51 proteins shared among the five patients were detected. Further, since we considered proteins with a 1.3-fold change (≧1.3-fold or <0.77-fold) for iTRAQ ratios as proteins with significant different levels [12, 21, 22], 13 of the 51 proteins in at least two samples of four volunteers' control sera for which iTRAQ ratios at the second time point (*T*2) to those at the first time point (*T*1) showed ≧1.3-fold or <0.77-fold difference (*T*2 versus *T*1) were excluded. Finally, among the 38 remaining proteins, the 16 proteins that show statistically significant levels of postsurgical/presurgical (patients' sera) compared with those of *T*2/*T*1 (control sera) (*P* < 0.05, patient versus control, Student's *t*-test) were identified and they are listed in the order of iTRAQ ratios in Table 3. In addition, we showed raw proteomic data of postsurgical versus presurgical sera in 5 CAS patients (P1~P5) in Supplementary Table  1 (in Supplementary Material available online at http://dx.doi.org/10.1155/2015/694120) and those of two time points (T2 versus T1) with a 14-day interval in 4 healthy volunteers (N1~N4) after FDR analyses in Supplementary Table  2.

### 3.2. Confirmation of the Accuracy of iTRAQ Ratios by Western Blot Analyses

**Figure 1:**
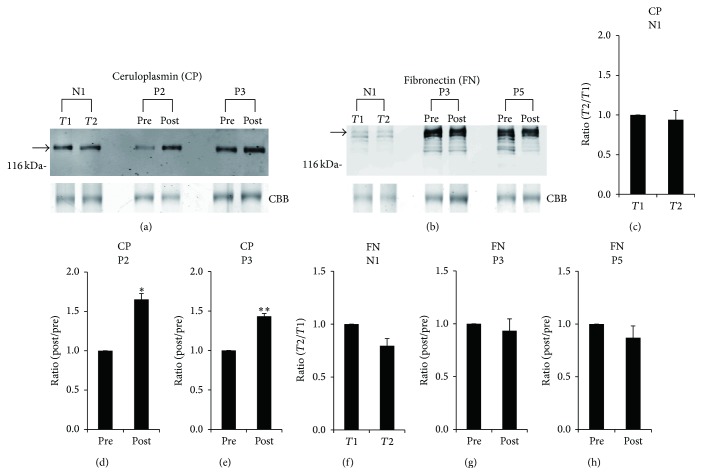
Verification of iTRAQ ratios by Western blot analyses. (a) Western blot analyses with anti-ceruloplasmin (CP) antibody in presurgical (pre) and postsurgical (post) sera of patients P2 and P3 as well as in sera of control volunteer N1 at two different time points (*T*1 and *T*2) were performed. (b) Western blot analyses with anti-fibronectin (FN) antibody in presurgical (pre) and postsurgical (post) sera of patients P3 and P5 were performed. Representative photos are shown. Some bands in the Western blot analysis with anti-FN antibody might be derived from proteolytic products of intact FN molecules. To confirm equal levels of proteins per lane, nonspecific proteins stained with Coomassie Brilliant Blue (CBB) are shown in the lowest panel. The intensity of each band that reacted with the corresponding antibody indicated by an arrow was measured. In the case of densitometric analysis of FN level, the intensity of the intact FN corresponding upper band was measured. Ratios of levels of ceruloplasmin in postsurgical sera to those in presurgical sera (1.0) in patient P2 (d) and patient P3 (e) were determined by band intensity. In addition, ratios of levels of fibronectin in postsurgical sera to those in presurgical sera in patient P3 (g) and patient P5 (h) were determined by band intensity. Similarly, ratios of levels of ceruloplasmin (c) and fibronectin (f) at *T*2 to those at *T*1 (1.0) in serum of control N1 were determined by band intensity. Means ± SE of triplicate experiments were calculated, and statistical analysis was performed using the paired* t*-test. ^*∗∗*^ *P* < 0.01. ^*∗*^ *P* < 0.05.

In order to confirm the accuracy of the quantitative results for the differentially expressed proteins that were identified, some proteins with an iTRAQ quantitative ratio were quantified again by Western blot analysis. The relative amounts of ceruloplasmin for patient P2 (iTRAQ ratio = 1.626) and patient P3 (1.444) and those of fibronectin for patient P3 (0.855) and patient P5 (0.825) were examined by Western blot analysis (Figures 1(a) and 1(b)). Subsequently, the ratios of levels of ceruloplasmin in postsurgical sera to those in presurgical sera (1.0) in patients P2 and P3 were determined by band intensity, and they were 1.653 for patient P2 (Figure 1(d)) and 1.433 for patient P3 (Figure 1(e)). On the other hand, those of fibronectin were 0.934 for patient P3 (Figure 1(g)) and 0.869 for patient P5 (Figure 1(h)). In the same way, iTRAQ ratios of ceruloplasmin (0.921) and fibronectin (0.761) in serum of a control volunteer N1 collected at two different time points (*T*1 and *T*2) were almost the same as the ratios determined by Western blot analysis (ceruloplasmin, 0.941; fibronectin, 0.795) (Figures 1(a), 1(b), 1(c), and 1(f)). These results indicated that iTRAQ ratios are almost consistent with quantitative results of Western blot analysis.

### 3.3. Classification of the 16 Identified Proteins with Differential Levels

As shown in Table 3, 15 of the 16 proteins were increased (≧1.0) and fibronectin was the only protein that was decreased (<1.0) in postsurgical sera compared with those in presurgical sera of patients with CAS. All of the proteins except for antithrombin-III, zinc-*α*-2-glycoprotein, and *α*-1B-glycoprotein were inflammatory response markers (positive acute-phase proteins) with increased levels during acute-phase response such as response to surgical invasion [23, 24]. In addition, fibronectin is known to be a negative acute-phase protein [25].

**Figure 2:**
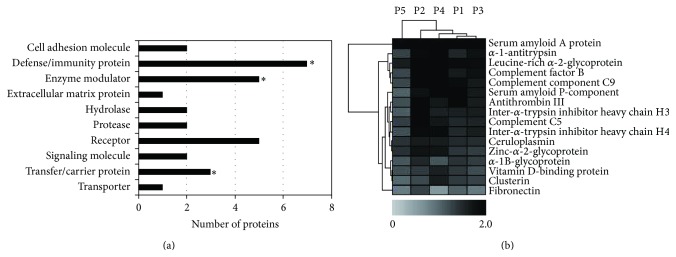
Panther protein class analysis (a) and hierarchical clustering analysis (b) of the 16 proteins with differential levels in presurgical and postsurgical sera of CAS patients. In (a), the stars indicate statistically significant classes (*P* < 0.05). In (b), differences in iTRAQ ratios are color-coded. Darker black spectra show more increased levels and lighter grey spectra show more decreased levels in postsurgical sera compared with those in presurgical sera.

For the functional distribution, the 16 identified proteins with differential levels in postsurgical and presurgical sera of patients with CAS were analyzed by the Panther classification, which sorts proteins into relevant classes based on their biological function (Figure 2(a)). By the statistical overrepresentation test in Panther, *α*-1-antitrypsin, antithrombin-III, inter-*α*-trypsin inhibitor heavy chain H3, complement C5, and inter-*α*-trypsin inhibitor heavy chain H4 were classified into enzyme modulator/serine protease inhibitor (*P* = 3.28 − *E*6), serum amyloid A protein, complement factor B, complement component C9, serum amyloid P component, complement C5, zinc-*α*-2-glycoprotein, and *α*-1B-glycoprotein were classified into defense/immunity protein (*P* = 3.02 − *E*5), and serum amyloid A protein, complement factor B, and complement component C9 were classified into transfer/carrier protein/apolipoprotein (*P* = 8.33 − *E*3).

Subsequently, we performed two-way hierarchical clustering analyses to evaluate expression patterns of the 16 proteins and relations of the 5 patients based on expression patterns of the 16 proteins. The results are shown in Figure 2(b).

### 3.4. Confirmation of Differential Levels of Antithrombin-III and Zinc-*α*-2-glycoprotein in Presurgical and Postsurgical Sera by Western Blot Analyses

**Figure 3:**
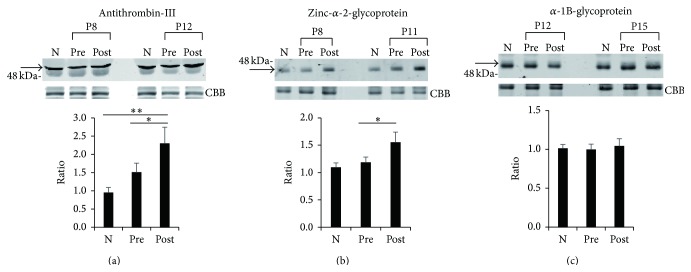
Increased levels of antithrombin-III and zinc-*α*-2-glycoprotein in postsurgical sera compared with those in presurgical sera of 14 CAS patients. Western blot analyses with anti-antithrombin-III antibody (a), anti-zinc-*α*-2-glycoprotein antibody (b), and anti-*α*-1B-glycoprotein antibody (c) in presurgical (pre) and postsurgical (post) sera of 14 patients (P6~P19) with CAS were performed. Arrow indicates reacted corresponding protein. Representative photos of two patients concerning each protein [patient numbers P8 and P12 for antithrombin-III in (a), P8 and P11 for zinc-*α*-2-glycoprotein in (b), and P12 and P15 for *α*-1B-glycoprotein in (c)] as well as a mixture (N) of 10 normal sera from control N5 to N14 are shown. To confirm equal levels of proteins per lane, nonspecific proteins stained with Coomassie Brilliant Blue are shown (CBB). The intensity of each band in presurgical and postsurgical sera of 14 patients and 10 normal control sera that reacted with each antibody was measured. Ratios of levels of each protein in presurgical and postsurgical sera from 14 patients and normal control sera from 10 volunteers compared with that in the mixture of 10 normal control sera (1.0) were determined by band intensity. Means ± SE of triplicate experiments were calculated, and statistical analysis between presurgical and postsurgical data was done with the paired* t*-test, whereas that between presurgical or postsurgical and normal control data was performed with the unpaired* t*-test. ^*∗∗*^ *P* < 0.01. ^*∗*^ *P* < 0.05.

It is conceivable that the three proteins, namely, antithrombin-III, zinc-*α*-2-glycoprotein, and *α*-1B-glycoprotein, besides acute-phase proteins among the 16 identified proteins are unique proteins with differential levels in presurgical and postsurgical sera of the CAS patients. To confirm this further, we performed Western blot analysis to determine the expression levels of antithrombin-III, zinc-*α*-2-glycoprotein, and *α*-1B-glycoprotein in postsurgical sera compared with the expression levels in presurgical sera from 14 other CAS patients (P6~P19). We confirmed increased levels of antithrombin-III and zinc-*α*-2-glycoprotein in postsurgical sera compared with those in presurgical sera of CAS patients (Figures 3(a) and 3(b)). Unfortunately, the levels of both antithrombin-III and zinc-*α*-2-glycoprotein in postsurgical sera were not similar to those in normal control sera. On the other hand, the expression level of *α*-1B-glycoprotein in postsurgical sera was not significantly different from that in presurgical sera from the 14 other CAS patients (Figure 3(c)). The results indicated that since the levels of antithrombin-III and zinc-*α*-2-glycoprotein in postsurgical sera did not return to levels similar to those in control sera, these two proteins are not candidates of disease markers for CAS but that they would become unique monitoring proteins for evaluating the pathophysiological and biochemical processes occurring before and after surgery for CAS.

### 3.5. Comparison of the 16 Differentially Expressed Proteins in Presurgical and Postsurgical Sera of CAS Patients with the 29 and 35 Differentially Expressed Proteins in Presurgical and Postsurgical Sera of Patients with Abdominal Aortic Aneurysm (AAA) and Thoracic Aortic Aneurysm (TAA), Respectively

**Figure 4:**
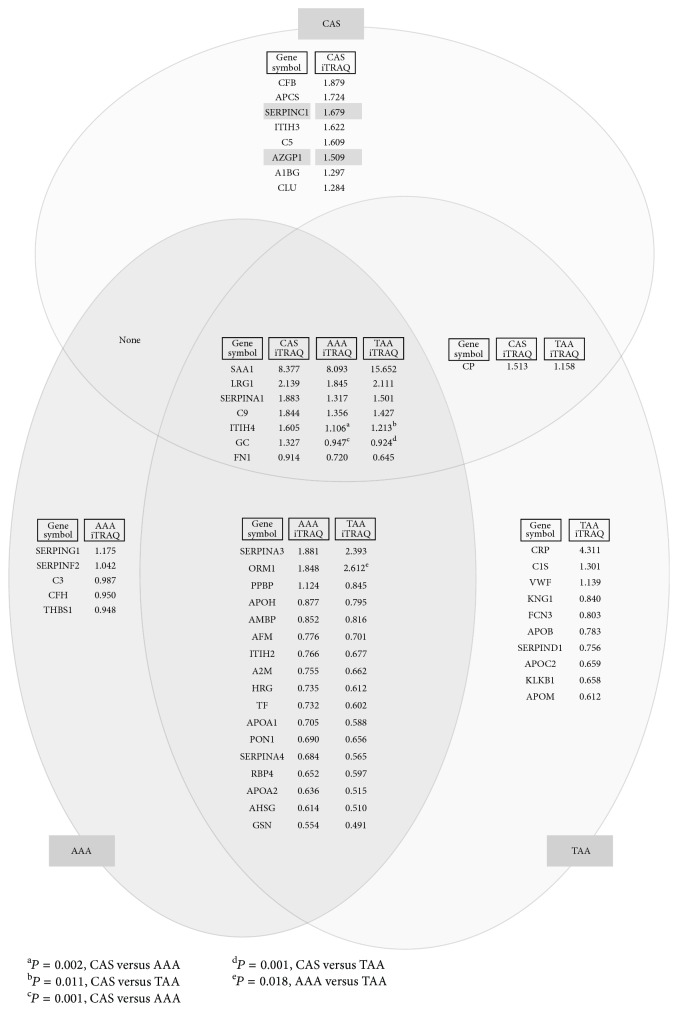
Venn diagrams showing proteins with differential levels in postsurgical and presurgical sera of patients with CAS, AAA, and TAA. The 29 and 35 serum proteins with differential levels in presurgical and postsurgical sera of AAA and TAA patients, respectively, identified in our previous study [12] were compared with the 16 differentially expressed proteins identified in presurgical and postsurgical sera of CAS patients. The Venn diagrams show inclusion and exclusion relations of datasets of each group with iTRAQ ratios (postsurgical versus presurgical) of the identified proteins. Antithrombin-III (SERPINC1) and zinc-*α*-2-glycoprotein (AZGP1) are emphasized by highlighter colors.

We previously reported the change in serum proteome before and after aortic aneurysm resection for patients with AAA and TAA using the same proteomic systems as that used in the present study [12]. In that study, 29 and 35 serum proteins were identified as proteins with differential levels in presurgical and postsurgical sera of AAA and TAA patients, respectively [12]. In the present study, we compared the 16 differentially expressed proteins in presurgical and postsurgical sera of CAS patients with the 29 and 35 differentially expressed proteins in presurgical and postsurgical sera of AAA and TAA patients, respectively, and we showed the results in Venn diagrams to visualize inclusion and exclusion relation of datasets of each group with iTRAQ ratios (postsurgical versus presurgical) (Figure 4). Interestingly, judging from the iTRAQ ratio of each protein, most of the acute-phase proteins shared among the three groups showed a tendency for the same increase and decrease. However, in 7 differentially expressed proteins shared among the sera of CAS, AAA, and TAA patients, quantitative differences of inter-*α*-trypsin inhibitor heavy chain H4 (gene name: ITIH4) and vitamin D-binding protein (GC) between presurgical and postsurgical sera of CAS patients were significantly distinct from those between presurgical and postsurgical sera of AAA and TAA patients (*P* < 0.05, ANOVA with post hoc by Scheffé's test). In addition, among the 17 differentially expressed proteins shared in the two groups of AAA and TAA patients except for proteins shared in all three groups, quantitative differences of *α*-1-acid glycoprotein (ORM1) were significantly distinct between AAA and TAA patients (*P* < 0.05, Student's *t*-test). Importantly, as expected, antithrombin-III (SERPINC1) and zinc-*α*-2-glycoprotein (AZGP1), possible monitoring proteins for surgery for CAS, were not included in the proteins that are differentially expressed in presurgical and postsurgical sera of AAA and TAA patients but were unique to those for CAS patients.

## 4. Discussion

In this study, we used a comprehensive proteomic approach for analyses of the dynamic changes in serum proteome before and after aortic valve replacement surgery for CAS patients. Sixteen serum proteins with differential levels in postsurgical and presurgical sera, most of which were nonspecific acute-phase proteins, were finally identified by the proteomic analyses. However, the remaining antithrombin-III, zinc-*α*-2-glycoprotein, and *α*-1B-glycoprotein were not nonspecific acute-phase proteins, and they seem to be differentially expressed proteins that increased exclusively after the surgery for CAS patients, since these three proteins were not identified as differentially expressed proteins in presurgical and postsurgical sera of AAA and TAA patients with aortic aneurysm resection as shown in Figure 4. In particular, antithrombin-III and zinc-*α*-2-glycoprotein were increased after surgery for the other 14 CAS patients. These results indicated that antithrombin-III and zinc-*α*-2-glycoprotein would become unique monitoring proteins for evaluating the biological responses occurring before and after surgery for CAS.

Antithrombin-III, which belongs to the family of serpins, plays a role in controlling the process of coagulation. It is an important inhibitor of coagulation proteases, such as thrombin and factor Xa [26]. Thus, inherited antithrombin-III deficiency causes venous thromboembolic disease. In addition to its role in hemostasis, there is accumulating evidence that antithrombin-III has anti-inflammatory actions [27]. On the other hand, zinc-*α*-2-glycoprotein, also known as a lipid-mobilizing factor, is involved in lipolysis [28]. Zinc-*α*-2-glycoprotein-deficient mice showed increased body weight compared with wild-type mice [29]. Apart from lipolytic function and adipose tissue atrophy, zinc-*α*-2-glycoprotein seems to be also involved in anti-inflammatory action [30]. In hemodialysis patients, the level of zinc-*α*-2-glycoprotein was shown to be negatively correlated with proinflammatory and proatherogenic markers including TNF-*α* and VCAM-1 [31]. It is known that the plasma level of thrombin-antithrombin-III complex (TAT) is significantly increased in patients with valvular heart diseases including mitral stenosis [32] and aortic stenosis [33], atrial fibrillation [34], and idiopathic cardiomyopathy [35], implying a state of increased coagulability in these heart diseases. In addition, it has been reported that serum zinc-*α*-2-glycoprotein levels are increased in advanced heart failure patients [36]. In line with this evidence, the present study demonstrated that the levels of antithrombin-III and zinc-*α*-2-glycoprotein in presurgical sera of CAS patients were higher than those in control sera as shown in Figure 3. Although we showed increased levels of antithrombin-III and zinc-*α*-2-glycoprotein in postsurgical sera compared with those in presurgical sera of CAS patients, the reason for the increase in levels of antithrombin-III and zinc-*α*-2-glycoprotein after surgery for CAS is not clear. However, as mentioned above, antithrombin-III and zinc-*α*-2-glycoprotein might display anti-inflammatory actions through an interaction with cells such as endothelial cells and leukocytes, leading to reduced expression of proinflammatory cytokines and diminished leukocyte activation. Presurgery and postsurgery for CAS would be under severe inflammatory conditions, and thus antithrombin-III and zinc-*α*-2-glycoprotein would be particularly upregulated to reduce aortic valve failure and tissue damage in the postoperative state.

Unfortunately, postsurgical levels of antithrombin-III and zinc-*α*-2-glycoprotein were not restored to the same levels as those in normal control sera after the surgery for CAS, suggesting that antithrombin-III and zinc-*α*-2-glycoprotein cannot be candidates of biomarkers for CAS. Sainger et al. [8] reported increases in some biomarkers including osteopontin, parathyroid hormone, fetuin-A, NT-proBNP, and ADMA for CAD progression. Among them, in the present study, only fetuin-A remained in the 38 penultimate proteins, but since the iTRAQ ratio of fetuin-A was not a statistically significant level (*P* = 0.812, patient versus control) (data not shown), the protein was eventually removed, leaving the final 16 proteins (see Section 3.1). Thus, these candidate biomarkers associated with CAD could not be identified in the present study. This might be due to differences in analytical methodologies that were used in the study by Sainger et al. [8] and our study for identification of biomarkers. Sainger et al. [8] examined candidate biomarkers based on previous studies, namely, principally using enzyme-linked immunosorbent assay (ELISA) in their case-control study.

Treatment of CAS includes the use of medical therapy such as renin-angiotensin inhibitors, diuretics, statins, and antiosteoporosis drugs. Furthermore, three of the five patients whose serum samples were used for proteomic analysis in this study also suffered from complications or received medications associated with calcium and phosphorus metabolism that might lead to ectopic calcification. In addition, 14 other patients whose serum samples were used for Western blot analysis also had these complications and medications without a significance difference compared with the five patients whose serum samples were used for proteomic analysis. Therefore, although potential effects of these complications and medications on presurgical and postsurgical proteomes cannot be ruled out, we speculate that the results obtained from analyses of the 19 CAS patients' sera are typical results for sera of the majority of CAS patients with aortic valve replacement surgery.

We propose that antithrombin-III and zinc-*α*-2-glycoprotein are unique monitoring proteins for evaluating the pathophysiological and biochemical processes occurring before and after surgery for CAS. Further additional studies, especially with biochemical analyses, are needed to elucidate the pathogenic roles of antithrombin-III and zinc-*α*-2-glycoprotein in the development and progression of CAS.

## 5. Conclusion

Proteome analyses of sera before and after aortic valve replacement surgery for CAS were performed with iTRAQ labeling followed by nanoLC-MALDI-TOF/TOF-MS/MS analysis, and changes in protein expression levels were verified by Western blot analyses. Among the identified proteins with differential levels before and after the surgery, the levels of antithrombin-III and zinc-*α*-2-glycoprotein were increased exclusively in postsurgical sera of CAS patients. Furthermore, according to our previous data on proteome changes in presurgical and postsurgical sera of aortic aneurysm patients, antithrombin-III and zinc-*α*-2-glycoprotein were not identified as differentially expressed proteins in presurgical and postsurgical sera of AAA and TAA patients. Our results provide useful information on unique monitoring proteins for assessing the pathophysiological processes occurring before and after surgery for CAS. The results should help to further clarify the cause and effect of CAS and contribute to the development of novel therapeutic strategies for CAS.

## Supplementary Material

We showed raw proteomic data of postsurgical versus presurgical sera in 5 CAS patients (P1-P5) in Supplementary Table 1 and those of two time points (T2 versus T1) with a 14-day interval in 4 healthy volunteers (N1-N4) after FDR analyses in Supplementary Table 2.

## Figures and Tables

**Table 1:** Comorbidities restricted to cardiovascular diseases, lipid metabolism disorders, and calcium and phosphorus metabolism disorders for the 19 CAS patients.

	Number (%)
	(*n* = 19)
Cardiovascular disorder	
Atrial fibrillation	4 (21.1)
Bicuspid aortic valve	2 (10.5)
Complete AV block	1 (5.3)
Congestive heart failure	5 (26.3)
Heyde's syndrome	1 (5.3)
History of stroke	1 (5.3)
Hypertension	9 (47.4)
Mitral valve disease	2 (10.5)
Peripheral arterial disease	1 (5.3)
Postoperative state of carotid artery stenting	1 (5.3)
Postoperative state of coronary stent implantation	2 (10.5)
Postoperative state of mitral valve replacement	1 (5.3)
Postoperative state of pacemaker implantation	1 (5.3)
Tricuspid valve regurgitation	2 (10.5)

Lipid metabolism	
Dyslipidemia	7 (36.8)

Calcium and phosphorus metabolism	
Calcification	
Chronic kidney disease	6 (31.6)
Nephrogenic anemia	2 (10.5)
Osteoporosis	2 (10.5)

**Table 2:** Prescribed drugs restricted to cardiovascular disorder, blood coagulation, lipid metabolism, and calcium and phosphorus metabolism for the 19 CAS patients.

	Number (%)
	(*n* = 19)
Cardiovascular disorder	
ACE inhibitor	3 (15.8)
ARB	4 (21.1)
*α*1 blocker	1 (5.3)
Antiarrhythmic drug	1 (5.3)
Ca blocker	8 (42.1)
Diuretics	6 (31.6)
Kallidinogenase (drug for Meniere disease)	1 (5.3)
Nicorandil	1 (5.3)
Nitrate (isosorbide mononitrate)	1 (5.3)

Blood coagulation	
Anticoagulant (warfarin)	4 (21.1)
Antiplatelet drug	5 (26.3)
Dipyridamole	2 (10.5)

Lipid metabolism	
Statin	9 (47.4)

Calcium and phosphorus metabolism	
Calcification	
Bisphosphonate	3 (15.8)
Cinacalcet (antihyperparathyroidism drug)	2 (10.5)
Lanthanum carbonate hydrate (drug for hyperphosphatemia)	1 (5.3)
Precipitated calcium carbonate (drug for hyperphosphatemia)	1 (5.3)
Vitamin D (alfacalcidol)	1 (5.3)

ACE inhibitor, angiotensin-converting enzyme inhibitor; *α*1 blocker, alpha-1 blocker; ARB, angiotensin II receptor blocker; Ca blocker, calcium channel blocker (CCB).

**Table 3:** Proteins with differential levels in postsurgical and presurgical sera of the CAS patients.

Unused ProtScore^a^	%Coverage^b^	Peptides^c^ (95%)	UniProt number	Gene symbol	Protein name	iTRAQ ratio^d^ Average ± SE	*P* value^e^	Molecular function
14.0	76.2	10	P02735	SAA1	Serum amyloid A protein	8.377 ± 1.656	—^f^	Acute-phase reactant
18.0	40.4	9	P02750	LRG1	Leucine-rich *α*-2-glycoprotein	2.139 ± 0.224	0.0105	Unknown
83.2	73.7	93	P01009	SERPINA1	*α*-1-antitrypsin	1.883 ± 0.290	0.0289	Protease inhibitor
46.7	52.2	30	P00751	CFB	Complement factor B	1.879 ± 0.219	0.0136	Complement
12.2	17.2	7	P02748	C9	Complement component C9	1.844 ± 0.191	0.0176	Complement
13.4	35.4	10	P02743	APCS	Serum amyloid P component	1.724 ± 0.195	—^f^	Acute-phase reactant
38.3	52.8	19	P01008	SERPINC1	Antithrombin-III	1.679 ± 0.146	0.0060	Blood coagulation
11.0	19.0	7	Q06033	ITIH3	Inter-*α*-trypsin inhibitor heavy chain H3	1.622 ± 0.189	—^f^	Hyaluronan binding
19.7	14.1	9	P01031	C5	Complement C5	1.609 ± 0.120	0.0042	Complement
39.0	33.9	24	Q14624	ITIH4	Inter-*α*-trypsin inhibitor heavy chain H4	1.605 ± 0.132	0.0152	Acute-phase reactant
116.0	61.6	72	P00450	CP	Ceruloplasmin	1.513 ± 0.045	0.0212	Transporter
32.4	57.3	21	P25311	AZGP1	Zinc-*α*-2-glycoprotein	1.509 ± 0.087	0.0275	Lipid catabolism
51.0	69.8	25	P02774	GC	Vitamin D-binding protein	1.327 ± 0.075	0.0435	Sequestration of actin
27.6	40.4	13	P04217	A1BG	*α*-1B-glycoprotein	1.297 ± 0.078	0.0236	Unknown
27.1	38.8	17	P10909	CLU	Clusterin	1.284 ± 0.100	0.0390	Extracellular chaperone
71.3	28.0	35	P02751	FN1	Fibronectin	0.914 ± 0.116	—^f^	Extracellular matrix

^a^A score of protein confidence (ProtScore) for a detected protein that is calculated from the peptide confidence from spectra that are not already “used” by higher scoring proteins in the experiments.

^b^The percentage of matching amino acids from identified peptides.

^c^The number of distinct peptides with at least 95% confidence in the experiments.

^d^iTRAQ ratio of postsurgical sera compared with those in presurgical sera (postsurgical versus presurgical).

^e^Statistical analysis of iTRAQ ratio of postsurgery/presurgery of patients' sera compared with that of *T*2/*T*1 of control volunteers' sera (patient versus control, Student's *t*-test).

^f^Protein that did not appear in more than two control sera.
